# Supplementary material for: A qualitative study of lived experiences of underrepresented electrical workers using creative non-fiction
Source: PLoS One. 2026 Mar 23;21(3):e0345572. doi: 10.1371/journal.pone.0345572 (PMC13008067; doi:10.1371/journal.pone.0345572)
Supplement: Attachment — Submitted filename: PONE-D-25-55981.docx [file pone.0345572.s001.docx]

PLOS ONE

A qualitative study of lived experiences of underrepresented electrical workers using creative non-fiction

--Manuscript Draft--

| Manuscript Number: | PONE-D-25-55981 |
| --- | --- |
| Article Type: | Research Article |
| Full Title: | A qualitative study of lived experiences of underrepresented electrical workers using creative non-fiction |
| Short Title: | Lived experiences of underrepresented electrical workers |
| Corresponding Author: | Behdin Nowrouzi-Kia  University of Toronto Temerty Faculty of Medicine Toronto, Ontario CANADA |
| Keywords: | Skilled trades, Gender equality, Mental health and well-being, Workplace integration, Qualitative methods |
| Abstract: | Objectives: Women, Indigenous peoples, racialized individuals, and persons with disabilities remain underrepresented in the electrical industry. This study explored the lived experience of underrepresented electrical workers related to their mental health and workplace integration.  Methods: A qualitative narrative design was employed. One-on-one interviews were conducted with eleven participants who self-identified as women, Indigenous peoples, racialized individuals, and/or persons with disabilities. Interview data were first analyzed using a narrative thematic approach and informed the development of creative non-fictional stories.  Results: Three stories were developed. Story 1 – “Asking for a ride: being a women electrician” illustrated the experiences of a woman apprentice who faced inadequate job site accommodations and sexism challenges in the workplace. Story 2 – “The lunch talk: Indigenous people and racilized individuals in the trade” highlighted the experiences of Indigenous and racialized participants who encountered language barriers and discriminatory comments. Story 3 – “Luke’s notes: living and working with disability” demonstrated the impacts of physical disability on the daily work of electrical workers, particularly in managing the physical demands and mental health strains. Conclusions: Electrical workers from underrepresented groups experience persistent barriers to mental health and workplace integration, including a lack of accommodations, limited social support, and experiences of discrimination at the workplace. These individuals also reported challenges in seeking workplace support due to a “toughness” culture within the industry. Electrical employers should foster an inclusive organizational culture that prioritizes the health and psychosocial well-being of underrepresented workers. |
| Order of Authors: | Zhiyang Shi, PhD |
|  | Donia Obeidat, MSc |
|  | Ali Bani-Fatemi, PhD |
|  | Aaron Howe, MA |
|  | Behdin Nowrouzi-Kia, PhD |
| Additional Information: |  |
| Question | Response |
| Financial Disclosure  Enter a financial disclosure statement that describes the sources of funding for the work included in this submission. Review the [submission guidelines](http://journals.plos.org/plosone/s/submission-guidelines#loc-financial-disclosure-statement) for detailed | Yes |

| requirements. View published research articles from [PLOS ONE](https://journals.plos.org/plosone/) for specific examples.  This statement is required for submission and will appear in the published article if the submission is accepted. Please make sure it is accurate. |  |
| --- | --- |
| Funded studies Enter a statement with the following details: - Initials of the authors who received each award - Grant numbers awarded to each author - The full name of each funder - URL of each funder website - Did the sponsors or funders play any role in the study design, data collection and analysis, decision to publish, or preparation of the manuscript? |  |
| Did you receive funding for this work? |  |
| Please add funding details.   as follow-up to "Financial Disclosure  Enter a financial disclosure statement that describes the sources of funding for the work included in this submission. Review the [submission guidelines](http://journals.plos.org/plosone/s/submission-guidelines#loc-financial-disclosure-statement) for detailed requirements. View published research articles from [PLOS ONE](https://journals.plos.org/plosone/) for specific examples.  This statement is required for submission and will appear in the published article if the submission is accepted. Please make sure it is accurate. | ZS received financial support from the Ontario Electrical League (https://www.oel.org/). Grant number is not applicable. The Ontario Electrical League contributed to the study conceptualization, recruitment, and data collection. |

| Funded studies Enter a statement with the following details: - Initials of the authors who received each award - Grant numbers awarded to each author - The full name of each funder - URL of each funder website - Did the sponsors or funders play any role in the study design, data collection and analysis, decision to publish, or preparation of the manuscript? |  |
| --- | --- |
| Did you receive funding for this work?" |  |
| Please select the country of your main research funder (please select carefully as in some cases this is used in fee calculation).   as follow-up to "Financial Disclosure  Enter a financial disclosure statement that describes the sources of funding for the work included in this submission. Review the [submission guidelines](http://journals.plos.org/plosone/s/submission-guidelines#loc-financial-disclosure-statement) for detailed requirements. View published research articles from [PLOS ONE](https://journals.plos.org/plosone/) for specific examples.  This statement is required for submission and will appear in the published article if the submission is accepted. Please make sure it is accurate. | CANADA - CA |
| Funded studies Enter a statement with the following details: - Initials of the authors who received each award - Grant numbers awarded to each author - The full name of each funder - URL of each funder website - Did the sponsors or funders play any role in the study design, data collection and analysis, decision to publish, or preparation of the manuscript? |  |
| Did you receive funding for this work?" |  |
| Competing Interests | The authors have declared that no competing interests exist. |

| Use the instructions below to enter a competing interest statement for this submission. On behalf of all authors, disclose any [competing interests](http://journals.plos.org/plosone/s/competing-interests) that could be perceived to bias this work—acknowledging all financial support and any other relevant financial or nonfinancial competing interests.  This statement is required for submission and will appear in the published article if the submission is accepted. Please make sure it is accurate and that any funding sources listed in your Funding Information later in the submission form are also declared in your Financial Disclosure statement.  View published research articles from [PLOS ONE](http://journals.plos.org/plosone/) for specific examples. |  |
| --- | --- |
| NO authors have competing interests Enter: The authors have declared that no competing interests exist. Authors with competing interests Enter competing interest details beginning with this statement: I have read the journal's policy and the authors of this manuscript have the following competing interests: [insert competing interests here] |  |
| * typeset |  |
| Ethics Statement  Enter an ethics statement for this submission. This statement is required if the study involved:   - Human participants - Human specimens or tissue - Vertebrate animals or cephalopods - Vertebrate embryos or tissues | This research has been approved by the Office of Research Ethics at University of Toronto [#41194]. All participants provided informed written informed consent prior to participation. The study did not include minors. |

| • Field research  Write "N/A" if the submission does not require an ethics statement.  General guidance is provided below. Consult the [submission guidelines](http://journals.plos.org/plosone/s/submission-guidelines#loc-guidelines-for-specific-study-types) for detailed instructions. Make sure that all information entered here is included in the Methods section of the manuscript. |  |
| --- | --- |

Format for specific study types

Human Subject Research (involving human

participants and/or tissue)

Give the name of the institutional review

board or ethics committee that approved the

study

•

Include the approval number and/or a

statement indicating approval of this

research

•

Indicate the form of consent obtained

(

written/oral) or the reason that consent was

not obtained (e.g. the data were analyzed

anonymously)

•

Animal Research (involving vertebrate

animals, embryos or tissues)

Provide the name of the Institutional Animal

Care and Use Committee (IACUC) or other

relevant ethics board that reviewed the

study protocol, and indicate whether they

approved this research or granted a formal

waiver of ethical approval

•

Include an approval number if one was

obtained

•

If the study involved

non-human primates

,

add

additional details

about animal welfare

and steps taken to ameliorate suffering

•

If anesthesia, euthanasia, or any kind of

animal sacrifice is part of the study, include

briefly which substances and/or methods

were applied

•

Field Research

Include the following details if this study

involves the collection of plant, animal, or

other materials from a natural setting:

Field permit number

•

Name of the institution or relevant body that

granted permission

•

Data Availability

Authors are required to make all data

underlying the findings described fully

available, without restriction, and from the

time of publication. PLOS allows rare

exceptions to address legal and ethical

concerns. See the

[PLOS Data Polic](http://journals.plos.org/plosone/s/data-availability)

[y](http://journals.plos.org/plosone/s/data-availability)

and

[FA](http://journals.plos.org/plosone/s/data-availability#loc-faqs-for-data-policy)

[Q](http://journals.plos.org/plosone/s/data-availability#loc-faqs-for-data-policy)

for detailed information.

No - some restrictions will apply

| A Data Availability Statement describing where the data can be found is required at submission. Your answers to this question constitute the Data Availability Statement and will be published in the article, if accepted. |  |
| --- | --- |
| Important: Stating ‘data available on request from the author’ is not sufficient. If your data are only available upon request, select ‘No’ for the first question and explain your exceptional situation in the text box. |  |
| Do the authors confirm that all data underlying the findings described in their manuscript are fully available without restriction? |  |
| Describe where the data may be found in full sentences. If you are copying our sample text, replace any instances of XXX with the appropriate details.   - If the data are held or will be held in a public repository, include URLs, accession numbers or DOIs. If this information will only be available after acceptance, indicate this by ticking the box below. For example: All XXX files   are available from the XXX database (accession number(s) XXX, XXX.).   - If the data are all contained within the manuscript and/or Supporting Information files, enter the following: All relevant data are within the manuscript and its Supporting Information files. - If neither of these applies but you are able to provide details of access elsewhere, with or without limitations, please do so. For example:   Data cannot be shared publicly because of [XXX]. Data are available from the XXX Institutional Data Access / Ethics Committee (contact via XXX) for researchers who meet the criteria for access to confidential data.  The data underlying the results presented in the study are available from (include the name of the third party | Data cannot be shared publicly because of the privacy and confidentiality policy of the  University of Toronto. Data are available from the University of Toronto's Institutional Data Access / Ethics Committee (contact via the corresponding author) for researchers who meet the criteria for access to confidential data. |

| and contact information or URL).  • This text is appropriate if the data are owned by a third party and authors do not have permission to share the data.  * typeset |  |
| --- | --- |
| Additional data availability information: |  |

Manuscript	[Click here to](https://www2.cloud.editorialmanager.com/pone/download.aspx?id=42494989&guid=63ea3ed4-704c-45bf-a941-613b1040136c&scheme=1)

[access/download;Manuscript;2025_OEL_QUAL_PLOS](https://www2.cloud.editorialmanager.com/pone/download.aspx?id=42494989&guid=63ea3ed4-704c-45bf-a941-613b1040136c&scheme=1)

# 1 A qualitative study of lived experiences of underrepresented electrical workers using 2 creative non-fiction

3	Zhiyang Shi, PhD^1^, Donia Obeidat, M.Sc.^1,5,6^, Ali Bani-Fatemi, PhD^1^, Aaron Howe, M.A.^1^, 4	Behdin Nowrouzi-Kia, PhD^1,2,3,4^

5

6	1. Restore Lab, Department of Occupational Science and Occupational Therapy, Temerty 7	Faculty of Medicine, University of Toronto, Ontario, Canada M5G 1V7

8	2. Krembil Research Institute-University Health Network, 60 Leonard Ave, Toronto, 9	Ontario, Canada M5T 0S8

10	3. Institute for Mental Health Policy Research, Centre for Addiction and Mental Health, 11	1000 Queen St W, Toronto, Ontario, Canada M6J 1H4

12	4. Centre for Research in Occupational Safety & Health, Laurentian University, 935 13	Ramsey Lake Rd, Sudbury, Ontario, Canada P3E 2C6

14 5. Rehabilitation Science Institute, Temerty Faculty of Medicine, University of Toronto,
15 Toronto, Ontario, Canada M5G 1V7
16 6. Department of Rehabilitation Sciences, Faculty of Applied Medical Sciences, Jordan
17 University of Science and Technology, Irbid, Jordan

18

19 **Corresponding author:**
20 Behdin Nowrouzi-Kia, OT Reg. (Ont.), PhD
21 Department of Occupational Science and Occupational Therapy
22 160-500 University Ave. Toronto, Ontario, Canada M5G 1V7
23 Faculty of Medicine, University of Toronto, Toronto, Ontario, Canada
24 E-mail: behdin.nowrouzi.kia@utoronto.ca
25 ORCiD : 0000-0002-5586-4282

# Abstract

**Objectives:** Women, Indigenous peoples, racialized individuals, and persons with disabilities remain underrepresented in the electrical industry. This study explored the lived experience of underrepresented electrical workers related to their mental health and workplace integration.

**Methods:** A qualitative narrative design was employed. One-on-one interviews were conducted with eleven participants who self-identified as women, Indigenous peoples, racialized individuals, and/or persons with disabilities. Interview data were first analyzed using a narrative thematic approach and informed the development of creative non-fictional stories.

**Results:** Three stories were developed. Story 1 – “Asking for a ride: being a women electrician” illustrated the experiences of a woman apprentice who faced inadequate job site accommodations and sexism challenges in the workplace. Story 2 – “The lunch talk: Indigenous people and racilized individuals in the trade” highlighted the experiences of Indigenous and racialized participants who encountered language barriers and discriminatory comments. Story 3 – “Luke’s notes: living and working with disability” demonstrated the impacts of physical disability on the daily work of electrical workers, particularly in managing the physical demands and mental health strains.

**Conclusions:** Electrical workers from underrepresented groups experience persistent barriers to mental health and workplace integration, including a lack of accommodations, limited social support, and experiences of discrimination at the workplace. These individuals also reported challenges in seeking workplace support due to a “toughness” culture within the industry. Electrical employers should foster an inclusive organizational culture that prioritizes the health and psychosocial well-being of underrepresented workers.

# INTRODUCTION

Equity-deserving groups including women, Indigenous peoples, racialized individuals, and persons with disabilities are underrepresented in the Candian skilled trades. Women make up merely 7.4% of the workforce [1], Indigenous peoples account for 0.7% [2], racialized individuals comprise 21% [3], and persons with disabilities represent 6.7% [4]. These numbers fall below each group’s share of the overall population, highlighting a persistent gap in these individuals’ representation and integration in the workplace. In the electrical sector, diversity appears to be even lower [3].

Several factors influence workplace integration in the skilled trades. As one of the most physically demanding trades, electrical workers frequently report high levels of job-related stress and exposure to challenging work environments [5]. Social relationships with coworkers and employers also play a role in workplace engagement and job satisfaction [6]. The combined effects of physical demands and psychosocial stressors have been linked to poor mental health outcomes [7]. Despite these concerns, existing research has primarily focused on white, male workers, leaving a significant gap in understanding the experiences of women, Indigenous peoples, racialized individuals, and persons with disabilities.

Tradeswomen and racialized tradespeople reported lacking workplace support and encountering challenges such as job insecurity [7–10]. The lived experiences of Indigenous workers and persons with disabilities are particularly underresearched, despite the importance of these communities to the Canadian labour force. Furthermore, while these issues have been identified across the broader skilled trades, electrical workers may face unique challenges due to the high physical and cognitive job demands. For example, constant exposure to hazardous environments can lead to chronic fear, stress, and mental health strain [12–14]. These problems may be intensified by experiences of sexism, racism, and ableism within the workplace [15–17]. There is a need to investigate how underrepresented individuals navigate the intersection of physical, cognitive, and psychosocial stress [18].

The purpose of this study was to explore the lived experience of electrical workers who self-identify as women, Indigenous peoples, racialized individuals, and/or persons with disabilities, with particular attention to mental health and workplace integration. The research questions were: 1) What are the workplace experiences of electrical workers from underrepresented groups? and 2) What suggestions do these individuals have for promoting mental health and workplace integration?

**METHODS**

# Design and Philosophical Paradigm

This study employed a narrative design, which focuses on constructing meaning from human experiences through storytelling [19]. The approach aligned with our research objective to offer insight into individuals’ identities, values, and lived realities [20].

Our philosophical paradigm was interpretivist, meaning that we believed “reality is dependent on those interpreting it”, and meanings were generated “through transactions between researcher and participant, each bringing their prior experiences and understandings to bear on the topic of investigation” [21]. The research team comprised both university-based investigators with expertise in occupational health, psychology, and disability studies, and community partners from the electrical trades.

# Participants Recruitment

Participants included electrical apprentices, workers, and employers who self-identified as women, Indigenous peoples, racialized individuals, and/or persons with disabilities. Recruitment was conducted through convenience sampling in collaboration with the Ontario Electrical League, a provincial advocacy organization representing 38,000 non-unionized electrical workers. The Ontario Electrical League staff made initial contact with eligible participants and gauged their interest. With participants’ consent, the Ontario Electrical League shared contact information (participants’ email and/or phone number) with the research team. Additional recruitment was carried out at the Ontario Electrical League’s chapter meetings across Ontario. Recruitment took place between November 15, 2024 and March 31, 2025.

# Data Collection

First author (ZS) conducted one-on-one, semi-structured interviews with participants who provided informed consent. Each interview was audio-recorded and set to be within 30 minutes. In cases where the full interview guide (Appendix) was not covered in the initial session, a second interview was conducted. Up to two shorter interviews allowed for greater rapportbuilding, improved data richness, and respected participants' time, while enhancing reflexivity and trust [22]. Interviews were conducted online or by phone, based on participant preference and availability. All procedures were approved by the Univeristy of Toronto.

# Data Analysis

All interviews were transcribed and imported into NVivo 12 [23]. We started with a narrative thematic analysis to identify common ideas and threads across participants [24]. Following the methodological guideline [25], two researchers independently reviewed all transcripts and generated initial codes from each interview. These codes were then discussed between the coders until they reached agreement. This iterative coding process led to the developing of themes and sub-themes through collaborative reflection and questioning.

We used the thematic findings to develop creative non-fictional stories representing participants’ lived experiences [26]. Themes and sub-themes were mapped onto narrative structure elements (story beginning, middle, and ending) to form story proposals. Participant quotes were embedded in the proposals to maintain a clear connection between source data and narrative form. Using a combination of participant’s quotes and the researcher’s narrative writing, stories were developed through employing the writing techniques recommended in the literature, such as developing character [27]. The research team reviewed the story proposals and drafts, provided feedback, and refined the stories.

We ensured the rigor of the analysis through several strategies aligned with qualitative research standards. First, our data collection and analysis methods were coherent with our narrative design and interpretivist paradigm [21]. Second, we followed the established guideline for narrative thematic analysis and recommendations for creative non-fictional writing. Third, the involvement of two independent coders in the narrative thematic analysis process allowed multiple perspectives and interpretations to be acknowledged via investigator triangulation [28]. Fourth, the full research team developed and refined the stories, which acted as critical friends to ensure reflexivity [29]. The executive director of the Ontario Electrical League contributed to validating the stories as a community expert. Their input ensured the stories were credible and authentic, strengthening the trustworthiness of the findings [30].

# RESULTS

Eleven participants completed the interviews, representing a diverse sample of electrical workers, To preserve confidentiality given the small sample size, only an overall summary of participants characteristics is reported. Participants ranged in age from 20 to 67 years old (Mean=41.3), and included six men and five women. Eight were married or in common-law relationship, and three were single. Nine participants had completed college/university or higher education. On average, participants had worked as electricians for 9.7 years and reported working 37.7 hours per week.

Three creative non-fictional stories are developed based on the interview data, with each representing the experiences of women (story 1), Indigenous peoples and racialized individuals (story 2), and persons with disabilities (story 3). Participants quotes are presented in italicized text.

# Story 1: Asking for a ride: being a women electrician

Context: Natalie is a young woman apprentice who just started the fourth year of their apprenticeship.

When Natalie steps onto the job site, it’s freezing. Natalie slings her toolbelt and gets to work. An hour in, Natalie feels the familiar pinch in her bladder. She looks around, knowing what she won’t find: no portable toilet, no site facilities of any kind. She mutters: “*Being in the trades, a lot of the time you're working in fields in the middle of nowhere. If the guys have to go pee, they just go in the middle of the field, whatever. But for a girl, it's very, very difficult.”*

The only option is the coffee shop five minutes down the road. Natalie *is not covered under the company's insurance to drive the work van, so she has to rely on other people to drive her.* And asking for this help never sits right with her because it means admitting she’s different. And different, in the trades, so often means less than. “*In this job, it's mostly meant that the guys assume that I can't do something because I'm a woman,”* Natalie reflects, *“If I go to lift something heavy, they're like, ‘oh, no let me help you’, which is totally fine. It's nice that they do that, but sometimes I feel like I don't need their help to do some stuff.”*

However, the discomfort is getting worse. Natalie swallows her pride and walks up to one of the guy coworkers and asks: “Hey, mind driving me to the coffee shop?”

The guy replies: “Sure.”

In the van, Natalie breaks the silence: “You know, *I think women can be really hard on themselves whenever they start to think about wanting to be in the trade. When I first started, I know the guys were joking, but some of them said that I was a lesbian just because I was an electrician, which I'm not and they just assume that.”*

Her coworker nods and responds: “I heard you. This job’s never been an easy one. There was *one girl I went to school with, she must have been 24, skinny. She’s not going to be lifting heavy cable, but she is smart, doing all the calculations for voltage drop. I relied on her help to get through many things. She was truly intelligent, and I think she would be great in those crawl spaces, and I hope she does get a job. But I can imagine older electricians looking at her and saying, ‘oh she’s just a young girl, what can she know?’”*

Natalie exhales and says: “You are absolutely right. *At my old job honestly, I was very uncomfortable a lot of the time. The guys there got along great, but it was mostly awkward for me because you go to work and then they would hit on me all day. My old job, my foreman asked me to move in with him. I had to switch crews.”*

Back on site, Natalie reflects on the conversation they had in the car: “I appreciate what he just did and said. *Who you work for and who you work with makes a huge difference. I've been pretty lucky in the fact that if anybody makes a sexist comment, it's usually one of my colleagues that'll step forward and be like, ‘hey no, that's not OK’, before I can even say or do anything.”*

She picks up her tools again. “*I really wish that this company would hire more women, because at lunch break, it's boy talk. Sometimes it'd be nice to have women to hang out with. I find just generally being a woman, I'm not as close with them, like there’s a wall between us that will never be crossed.”* Natalie continues the thoughts, “*Some high schools have mechanic shops. If there was more of this, and there are more examples of people on television or in the celebrity world showing females in this type of trades, more women would be attracted and curious about it. If you don't see those examples that visibly, then you never think of that as your option.”*

# Story 2: The lunch talk: Indigenous people and racialized individuals in the trade

Context: Barry is a racial minority and a new immigrant to Canada. Barry works for Mike, who is an Indigenous person and owning a contracting business.

The sun casts long shadows across the job site as the crew gathers around for lunch.

Barry reaches for a sandwich and glances at his coworkers who are chatting easily in English. Laughter erupts at a joke, but Barry doesn’t get it. He just smiles anyway.

Mike, the owner of the company, notices Barry sitting a bit apart. He steps over and asks Barry: “How’s it going?”

Barry hesitates: “Good… I mean it just…” He pauses, searching for better words. “*Sometimes I still have a hard time explaining things to other guys. I just take it easy. It makes it easier to just write it… or to draw it down… what they need to do, instead of explaining it.”*

Mike smiles and responds: “The language, eh? That’s a big one.”

Barry stops chewing and says: “I also feel…outside.”

Mike sees the frustrations from Barry’s face. He leans closer and whispers to him: “I hear you. For me, it’s not the language, but being Indigenous, being a visible minority, the feeling of being on the outside never goes away. *I've had one really negative experience with a very racist customer, and that made me feel pretty awful. He was pretty racist and an anti-Indigenous person. My boss, who knows I'm an Indigenous person, kind of deflected the guy and redirected his attention. I just kept doing my thing and tried to ignore the gentlemen. I don’t think he was trying to be malicious, but I think he was a very old school, racist kind of dude. And it definitely was a shaking moment.”*

Barry nods and says: “Sorry to hear that.”

Mike speaks slowly: “Thanks. Before I started my own business, *I would be chosen for this one thing with one of the journeymen, and other people will be doing other things. I always wondered ‘why don’t they want me doing things on that team?’ And I would start to spin my wheels a little bit on that. Then I realized that the jobs were equally as important. I’m just working with this person versus that person.”*

The rest of the crew is picking back up. Mike talks to Barry: “*We all really benefit when we see diversity in any way. Diversity makes everyone feel more welcome. It benefits the whole company, homeowner and everyone. And that extrapolates up for so many different skills and experiences.”* Mike continues. “*There's been a lot of inclusiveness, not just in our trade, but for the other trades. Since now there's a lot of immigrants coming into the country, I don't know what religion they have, but people who wear turbans, some of the rules and the laws in the job site have been changed, because the hard hat does not fit over their head and their turban.”*

Mike claps Barry gently on the shoulder and says: “I’m glad to have you here. *I definitely try to make a point of hiring women and hiring people from underrepresented groups, because I've seen such value by being supported as one of those people in the trade.”*

Barry looks over at Mike, a quiet smile forming.

# Story 3: Luke’s notes: living and working with disability

Context: Luke is doing his second-year apprenticeship with a local contractor. He was diagnosed with a physical disability before becoming an electrical apprentice.

Bundles of wires lay across the job site. Luke takes a deep breath and pulls a notebook from his pocket. “Take list first”, he reminds himself, *“Before I had all this [disability], my memory was on point. I'd be able to remember measurements. Now I have to write it down on a piece of paper or on my phone, which kind of takes a little bit more time for me.”*

While the other apprentices are already running wire, Luke crouches beside the truck and carefully writes out his tasks. He tries to do it quickly but knows that his body works differently now. Luke mutters to himself: *“Through the disability, my bones hurt consistently all the time. It doesn't matter if I'm climbing a ladder or bending over to pick up a juice box or whatever. It’s the same amount of pain. I can be standing there and my body's aching 24/7.”*

Luke’s mind drifts back to when he was applying for this job, “*When I went to all the interviews, I didn't have [disability] on my resume, but it was something that I mentioned to them [employers] through that interview towards the end. In every job site I go on, whoever is in charge, I let them know because it's a safety thing for myself, and it's a safety thing for them, right? I don't try to let my disability to get into the way of my work. But at the end of the day, it's something that my employers got to accept.”* However, disclosing the disability was not an easy decision at that time. Luke thinks, “*To be honest, I was a little bit nervous that they [employers] would either look at me a different way or be like, ‘oh, we don't want to have a guy that misses days.’ Would I have a label put on me? Would my resume be tossed off to the side just because of the [disability]?”* His thoughts are interrupted as he wraps up his notes.

“Hey buddy, I need your help here!”, a coworker calls out.

“What’s up?”, Luke replies and heads over. He’s eager to support his coworkers - because he knows what it feels like to be supported. *When he told his bosses about his disability, they’ve always made sure that there’s somebody on sites who knows CPR.* Luke remembers one specific moment, “*Last summer we were doing the fire alarm testing. I don’t feel comfortable with all the strobe lights because of the [disability]. I told the head electrician and asked if he could find something else for me to do while all this testing was going around. He asked me to stay outside and let me know when to come back in. I definitely feel supported, because I would have a seizure if I stayed in the building, hypothetically.”* Thinking of that, Luke smiles. He feels ready for whatever the day brings.

# DISCUSSION

This study explored the lived experience of electrical workers from underrepresented groups. The narratives captured how participants navigated challenges related to mental health and workplace integration.

Story 1 illustrated the experiences of a woman apprentice who faced inadequate job site accommodations and sexism challenges. The severe underrepresentation of women in the electrical trades is compounded by the absence of basic supports, such as access to clean toilets and properly sized tools. Women participants also discussed how their relationships with employers and coworkers influenced their intention to remain or leave the job. They emphasized the necessity of receiving respect from man coworkers and experiencing a sense of relatedness with women coworkers. Similar to other trades [31], woman electricians were perceived as competent workers. Increasing the visibility of woman role models within the education system may help attract younger generations to pursue these careers.

Story 2 highlighted the experiences of Indigenous and racialized participants, who encountered language barriers and workplace racism. As Canada continues to welcome immigrants from non-English and non-French-speaking countries [32], language proficiency has become a critical determinant of professional success [33]. In this study, participants reported receiving little support from the employer. Given the collaboration nature of electrical work, employers need to offer support to workers who speak Indigenous or non-official languages, such as language training and employment counselling [34] Employers also have a responsibility to address racism and discrimination, as these issues continue to be prevalent across the skilled trades [35]. Although our findings suggested that overt incidents of discrimination were not frequent, their impact on participants’ emotional and psychosocial well-being was significant.	Story 3 demonstrated the impacts of living with a physical disability on the daily work, particularly in managing the physical demands and mental health strains. Individuals with disabilities are at greater risk for mental health conditions [36], and the physical and cognitive work demands may further exacerbate these issues. For example, participants reported experiencing heightened stress at job sites where the conditions could trigger symptoms related to their disability. We found that the participants’ decision to disclose was driven by their personal health and safety priority. However, such decisions can be particularly difficult for individuals who prioritize career development and fear potential negative consequences [37], as 35% of Canadian workers with disabilities never request accommodations from employers [38]. Inclusive workplace must encompass individual accommodations (e.g., modified work hours) and an organizational culture that prioritizes health, safety, and psychosocial well-being.

Across all three stories, participants described similar mental health challenges to those experienced by electrical workers more broadly, such as anxiety, stress, and burnout [7]. However, these underrepresented workers tended to rely on non-workplace support to cope with the challenges. They also acknowledged the presence of a “toughness” culture, which discouraged open discussion about mental health or limited their intention to seek support from the workplace. These findings emphasized the urgent need for cultural change within the industry, including refining the employer-employee hierarchy and increasing diversity in the workplace.

# Limitations

Each narrative reflects the experiences of individuals from a specific identity group; however, we did not examine the intersection between multiple underrepresented identities. Future research should adopt intersectional frameworks, such as Gender-Based Analysis Plus (GBA+), to understand better how overlapping identities shape workplace experiences. In addition, all participants were non-unionized workers, whose access to resources and support may differ from unionized counterparts. Expanding recruitment beyond the Ontario Electrical

League may provide a more comprehensive view of experiences across diverse work settings.

# Conclusion

Electrical workers from underrepresented groups, including women, Indigenous peoples, racialized individuals, and persons with disabilities, experience persistent barriers to mental health and workplace integration. Our study identified multiple key barriers, including a lack of accommodations, limited social support, and experiences of discrimination at the workplace. A “toughness” culture within the electrical industry has discouraged these individuals to seek workplace support. Employers in the electrical industry should foster an inclusive organizational culture that prioritizes the health and psychosocial well-being of underrepresented workers.

# Acknowledgements

We would like to express our gratitude to all research participants, the collaborators, Beatrice

Sharkey, Wendy Dobison and RoseMary MacVicar-Elliott from the Ontario Electrical League. Zhiyang Shi contributed to the conceptualization and design of the study, data collection, analysis, and interpretation of results, and the manuscript writing. Donia Obeidat contributed to the conceptualization and design of the study, data collection, analysis, and interpretation of results, and the manuscript writing. Ali Bani-Fatemi contributed to the conceptualization and design of the study, interpretation of results, and the manuscript writing. Aaron Howe contributed to the conceptualization and design of the study, and interpretation of results, and the manuscript writing. Behdin Nowrouzi-Kia contributed to the conceptualization and design of the study, data collection, analysis, and interpretation of results, and the manuscript writing. The data of this study are available from the corresponding author upon request. No AI was used at any stage during research development & design, data collection, manuscript preparation etc.

# References

1. Government of Canada SC. Proportion of women and men employed in occupations, annual, inactive [Internet]. 2019 [cited 2025 Jun 3]. Available from: https://www150.statcan.gc.ca/t1/tbl1/en/tv.action?pid=1410033502
2. Government of Canada SC. Employment by Indigenous group and occupation, inactive [Internet]. 2025 [cited 2025 Jun 3]. Available from:

https://www150.statcan.gc.ca/t1/tbl1/en/tv.action?pid=1410010401

3. Government of Canada SC. Visible minority by occupation, highest level of education and generation status: Canada, provinces and territories [Internet]. 2023 [cited 2025 Jun 3]. Available from: https://www150.statcan.gc.ca/t1/tbl1/en/tv.action?pid=9810033001
4. Government of Canada SC. Industry of employment for persons with and without disabilities aged 25 to 64 years, by sex [Internet]. 2020 [cited 2025 Jun 3]. Available from:

https://www150.statcan.gc.ca/t1/tbl1/en/tv.action?pid=1310075701

5. Nowrouzi-Kia B, Bani-Fatemi A, Howe A, Ubhi S, Morrison M, Saini H, et al. Examining burnout in the electrical sector in Ontario, Canada: A cross-sectional study. AIMS Public Health. 2023;10(4):934–51.
6. Bani-Fatemi A, Sanches M, Howe AS, Lo J, Jaswal S, Chattu VK, et al. Mental Health Outcomes among Electricians and Plumbers in Ontario, Canada: Analysis of Burnout and WorkRelated Factors. Behavioral Sciences. 2022 Dec;12(12):505.
7. Howe AS, Bani-Fatemi A, Tjahayadi E, Haritos A, Hao Y, Zhu S, et al. A quantitative examination of sleep quality, burnout, psychological distress, and social support availability of electrical workers in Ontario, Canada. Discov Public Health. 2024 Aug 12;21(1):55.
8. Curtis HM, Meischke H, Stover B, Simcox NJ, Seixas NS. Gendered Safety and Health Risks in the Construction Trades. Annals of Work Exposures and Health. 2018 Apr 18;62(4):404–15.
9. Norberg C, Johansson M. “Women and ‘Ideal’ Women”: The Representation of Women in the Construction Industry. Gend Issues. 2021 Mar 1;38(1):1–24.
10. Wang Y, Aminian E, Stewart I. Barriers Contributing to the Underrepresentation of Women in the Construction Industry. icgr. 2025 Apr 4;8(1):421–9.
11. Howe AS, Tan J, Yuen B, Saini H, Saade-Cleves N, Obeidat D, et al. Physical and Psychosocial Correlates of Occupational Physical Injury in the Global Construction Industry: A Scoping Review. Environ Health Insights. 2024;18:11786302241270371.
12. Mohammed Azeez, Gambatese J, Liu D. Comparison of Job Cognitive and Physical

Demands in Different Industries for Implications on Safety Outcomes. CSCE Annual Conference. 2019 Jun 12;

13. Singerman J, Gomez M, Fish JS. Long-Term Sequelae of Low-Voltage Electrical Injury. Journal of Burn Care & Research. 2008 Sep 1;29(5):773–7.
14. Thomée S, Jakobsson K. Life-changing or trivial: Electricians’ views about electrical accidents. WORK. 2018 Sep 18;60(4):573–85.
15. Grace SL, Tan Y, Cribbie RA, Nguyen H, Ritvo P, Irvine J. The mental health status of ethnocultural minorities in Ontario and their mental health care. BMC Psychiatry. 2016 Feb 26;16(1):47.
16. Lindsay S, Fuentes K, Tomas V, Hsu S. Ableism and Workplace Discrimination Among Youth and Young Adults with Disabilities: A Systematic Review. J Occup Rehabil. 2023 Mar;33(1):20–36.
17. Rotimi FE, Burfoot ,M., Naismith ,N., Mohaghegh ,M., and Brauner M. A systematic review of the mental health of women in construction: future research directions. Building Research & Information. 2023 May 19;51(4):459–80.
18. Bridges D, Wulff ,Elizabeth, Bamberry ,Larissa, Krivokapic-Skoko ,Branka, and Jenkins S. Negotiating gender in the male-dominated skilled trades: a systematic literature review.

Construction Management and Economics. 2020 Oct 2;38(10):894–916.

19. Bradbury-Jones C, Breckenridge ,Jenna, Clark ,Maria T., Herber ,Oliver R.,

Wagstaff ,Christopher, and Taylor J. The state of qualitative research in health and social science literature: a focused mapping review and synthesis. International Journal of Social Research Methodology. 2017 Nov 2;20(6):627–45.

20. Butina M. A Narrative Approach to Qualitative Inquiry. American Society for Clinical Laboratory Science. 2015 Jul 1;28(3):190–6.
21. Poucher ZA, Tamminen ,Katherine A., Caron ,Jeffrey G., and Sweet SN. Thinking through and designing qualitative research studies: a focused mapping review of 30 years of qualitative research in sport psychology. International Review of Sport and Exercise Psychology. 2020 Jan 1;13(1):163–86.
22. Goyes DR, Sandberg S. Trust, nuance, and care: Advantages and challenges of repeat qualitative interviews. Qualitative Research. 2025 Apr 1;25(2):330–49.
23. Jackson K, Bazeley P. QUALITATIVE DATA ANALYSIS WITH NVIVO. 3rd Edition.

SAGE Publications Inc.; 2019. 61 p.

24. Orr K, Smith ,Brett, Arbour-Nicitopoulos ,Kelly P., and Wright FV. The café talk: a discussion of the process of developing a creative non-fiction. Qualitative Research in Sport, Exercise and Health. 2021 Nov 2;13(6):887–903.
25. Castleberry A, Nolen A. Thematic analysis of qualitative research data: Is it as easy as it sounds? Curr Pharm Teach Learn. 2018 Jun;10(6):807–15.
26. Cavallerio F, editor. Creative Nonfiction in Sport and Exercise Research. New York: Routledge; 2021. 194 p.
27. Caulley DN. Making Qualitative Research Reports Less Boring: The Techniques of Writing Creative Nonfiction. Qualitative Inquiry. 2008 Apr 1;14(3):424–49.
28. Donkoh S, Mensah J. Application of triangulation in qualitative research. Quantitative and mixed-methods studies are more popular among Ghanaian researchers than qualitative studies The preference for quantitative and mixed-methods studies originates from the notion that these are more scientific and rigorous, and produce generalizable findings and conclusions [Internet]. 2023 Jan 11 [cited 2025 Jun 4];Volume 10(Issue 1). Available from: https://medcraveonline.com/JABB/JABB-10-00319.pdf
29. Ronkainen NJ, and Wiltshire G. Rethinking validity in qualitative sport and exercise psychology research: a realist perspective. International Journal of Sport and Exercise Psychology. 2021 Jan 2;19(1):13–28.
30. Hemming L, Pratt D, Bhatti P, Shaw J, Haddock G. Involving an individual with livedexperience in a co-analysis of qualitative data. Health Expectations. 2021;24(3):766–75.
31. Husam S, Laishram B, Johari S. Framework to Enhance Gender Inclusion of Workers in Construction Sites. Journal of Construction Engineering and Management. 2024 Apr 1;150(4):04024018.
32. Government of Canada SC. Labour force characteristics by immigrant status, annual, inactive [Internet]. 2025 [cited 2025 Jun 4]. Available from:

https://www150.statcan.gc.ca/t1/tbl1/en/tv.action?pid=1410008301

33. Mooten N. Racism, Discrimination and Migrant Workers in Canada: Evidence from the Literature [Internet]. 2022 [cited 2025 Jun 4]. Available from: https://www.canada.ca/en/immigration-refugees-citizenship/corporate/reportsstatistics/research/racism-discrimination-migrant-workers-canada-evidence-literature.html
34. Banerjee R, Reitz JG, Oreopoulos P. Do large employers treat racial minorities more fairly? A new analysis of Canadian field experiment data [Internet]. Toronto Metropolitan University; 2025 Feb [cited 2025 Jun 4]. Available from:

https://rshare.library.torontomu.ca/articles/report/Do_large_employers_treat_racial_minorities_m ore_fairly_A_new_analysis_of_Canadian_field_experiment_data/14638335/1

35. Roelofs C, Sprague-Martinez L, Brunette M, Azaroff L. A qualitative investigation of Hispanic construction worker perspectives on factors impacting worksite safety and risk. Environmental Health. 2011 Sep 30;10(1):84.
36. Koenig J, McLean KJ, Bishop L. Psychological distress and mental health diagnoses in adults by disability and functional difficulty status: Findings from the 2021 national health interview survey. Disability and Health Journal. 2024 Oct 1;17(4):101641.
37. von Schrader S, Malzer V, Bruyère S. Perspectives on Disability Disclosure: The Importance of Employer Practices and Workplace Climate. Employ Respons Rights J. 2014 Dec 1;26(4):237–55.
38. Government of Canada SC. Mental health-related disabilities, 2022 [Internet]. 2025 [cited 2025 Jun 4]. Available from: https://www150.statcan.gc.ca/n1/pub/11-627-m/11-627m2025010-eng.htm

Appendix_Interview Guide

[Click here to access/download](https://www2.cloud.editorialmanager.com/pone/download.aspx?id=42349152&guid=35221698-7331-4bf2-972f-72c5f67d3090&scheme=1)

[Supporting Information](https://www2.cloud.editorialmanager.com/pone/download.aspx?id=42349152&guid=35221698-7331-4bf2-972f-72c5f67d3090&scheme=1)

[Appendix copy.docx](https://www2.cloud.editorialmanager.com/pone/download.aspx?id=42349152&guid=35221698-7331-4bf2-972f-72c5f67d3090&scheme=1)

COREQ Checklist

[Click here to access/download Supporting Information](https://www2.cloud.editorialmanager.com/pone/download.aspx?id=42349151&guid=95ace2f5-6790-4a74-b378-7365e36fbd48&scheme=1)

[COREQ_Checklist_OEM copy.pdf](https://www2.cloud.editorialmanager.com/pone/download.aspx?id=42349151&guid=95ace2f5-6790-4a74-b378-7365e36fbd48&scheme=1)

Ethics Approval

[Click here to access/download](https://www2.cloud.editorialmanager.com/pone/download.aspx?id=42494881&guid=5f85ba47-2d84-429e-82ae-6482c7f7ae8d&scheme=1)

[Supporting Information SDF4_REB.pdf](https://www2.cloud.editorialmanager.com/pone/download.aspx?id=42494881&guid=5f85ba47-2d84-429e-82ae-6482c7f7ae8d&scheme=1)
