# Supplementary material for: A qualitative study of lived experiences of underrepresented electrical workers using creative non-fiction
Source: PLoS One. 2026 Mar 23;21(3):e0345572. doi: 10.1371/journal.pone.0345572 (PMC13008067; doi:10.1371/journal.pone.0345572)
Supplement: Attachment — Submitted filename: Response to Reviewers.docx [file pone.0345572.s002.docx]

**Response to Reviewers**

We truly appreciate your feedback on the manuscript. We have considered the additional comments from the reviewer and made modifications to the manuscript. We have outlined our responses and edits made below.

| Reviewer comment | Response |
| --- | --- |
| **Introduction** |  |
| Line 61-62: “Personally, whenever I'm reading about a disparity in representation I like to see the side by side of gen pop versus the area being examined” | Thank you for the suggestion. We have revised this sentence to:  “Despite representing 50.6% of the Canadian population, women make up merely 7.4% of the skilled trade workforce [1]. Indigenous peoples account for 0.7% of the workforce (vs. 5% of the general population) [2], racialized individuals comprise 21% (vs. 26.5%) [3], and persons with disabilities represent 6.7% (vs. 27%) [4]”  Line 61-65 |
| Line 77-79: “Are electrical workers an important group to study because of how they differ from other skilled trades? Or, how they are representative of the same issues? I'm not sure I understand from this intro why electricians face unique challenges.” | Thank you for the comment. According to Azeez et al. (2019), electrical workers are distinct from other skilled trades in its intersection of cognitive, physical, and safety demands. Azeez et al. (2019) identified electrical workers as having “the most cognitively demanding job” among the six trades examined. Other research also showed that electricians reported high levels of job-related stress and frequent exposure to hazardous work environments, such as electric shocks. Given these characteristics, electrical work represents a critical context for examining how these intense physical, cognitive, and psychosocial stressors intersect with lived experience of underrepresented workers. To make our rationale clearer, we have restructured the 2^nd^ and 3^rd^ paragraphs of the introduction:  “Several factors influence workplace integration in the skilled trades. For example, social relationships with coworkers and employers play an important role in workplace engagement and job satisfaction [5]. Tradeswomen and racialized tradespeople report lacking workplace support and encountering challenges such as job insecurity [6–9]. The lived experiences of Indigenous workers and persons with disabilities are particularly underresearched, despite the importance of these communities to the Canadian labour force.  Electrical work presents a distinct context within the skilled trades due to its intensive physical and cognitive demands. Compared to many other trades (e.g., carpenter), electrical work has been characterized as the most cognitively demanding trade due to its sustained vigilance and constant exposure to hazardous work environments, such as electrical shocks [10]. As a result, electrical workers frequently report high levels of job-related stress [11], chronic fear, and poor mental health outcomes [6, 12–14]. Despite these concerns, existing research has primarily focused on white, male electrical workers, leaving a significant gap in understanding the experiences of women, Indigenous peoples, racialized individuals, and persons with disabilities. Given that sexism, racism, and ableism may intensify these physical and cognitive demands [15-17], there is a need to investigate how underrepresented electrical workers navigate the intersection of these challenges [18].”  Line 68~84 |
| Methods |  |
| Line 106-110: What percentage of those eligible participated? Is there any particular reason to think those who responded are significantly different from those who didn't? | The partnering organization made initial contact with eligible participants and did not disclose the total number of individuals contacted. Therefore, we are unable to provide the percentage. It is possible that those who chose to participate were more actively engaged with the partnered organization, which may have influenced their experiences captured in this study. We have acknowledged this point as a study limitation.  “It was also possible that these participants were more actively engaged with the Ontario Electrical League. Expanding recruitment beyond the Ontario Electrical League and identifying less-active members may provide a more comprehensive view of experiences across diverse work settings.”  Line 396-399 |
| Line 126-127: This is admittedly the first time I've encountered this method. I would not be surprised if it's the same for other readers. As such, I think it's important to spend more time explaining this method, discussing previous publications that have used it, and generally proving it as a valid and worthwhile approach.  Again, take this with the necessary grain of salt as it may just be my own ignorance. | Thank you for raising this point. We agree that explaining the creative non-fiction method in more detail can enhance the clarity and transparency of our paper. We’ve made this addition as suggested:  “Creative non-fiction is a qualitative analytical practice used to represent participants’ lived experiences and researchers’ interpretations through a story-telling form [24]. It has been accepted as a valid and trustworthy method in sociological and psychological research, supported by the literature that provides systematic methodological guidance [27]. This method has been used to examine the lived experiences of underrepresented individuals in diverse contexts, including disability research [28].”  Line 161-168 |
| Results |  |
| Line 152-155: As I understand it, these stories are pieced together using quotes from multiple individuals. I'd be curious for each story to know how any individuals contributed. Or maybe, for each one to be labeled Participant 1, participant 2, etc.  Additionally, it was unclear to me if the overall plot of the story was something mentioned by any of the participants, if it emerged from overlapping themes, or if it was largely of your creation? This is particularly important to understand for the first story about needing a ride to the coffee shop to use the bathroom. | We appreciate your comments. We chose not to number or label individual participants due to the small sample size and the associated risk of participant identification. To enhance transparency while protecting confidentiality, we have instead indicated the number of participants whose quotes were included in each story:  “Story 1 incorporates quotes from five participants, story 2 has quotes from six participants, and story 3 includes quotes from one single participant. Quotes are not labeled by participant identifiers to reduce identification risk.”  Line 198-200  The overall plot of the story (e.g., a ride to the coffee shop to use the bathroom) was primarily constructed by the research team as part of the creative non-fiction writing process. It was not drawn verbatim from participant quotes. These scenario-based plots were to provide contextual coherence and facilitate narrative flow between quotes. This approach aligns with the established practices in creative non-fiction and has been used in prior work (e.g., Shi et al., 2024). To ensure methodological transparency, we’ve added this information to the methods section:  “Using a combination of participant’s quotes and the researcher’s narrative writing, stories were developed through employing the writing techniques recommended in the literature, such as establishing scenes, developing characters, and showcasing dialogues [29]. Scenario-based story plots were primarily constructed by the research team through creative writing to establish contextual coherence for each story.”  Line 170-174 |
| Line 267-268: I feel like all three stories should and hopefully could expanded to be much longer. As they stand, it felt primarily like a way to highlight some important quotes from your interviews. It didn't necessarily feel like a cohesive narrative. | Thank you for this thoughtful comment. The length of the stories was intentionally designed to foreground participants’ voices. A substantial proportion of each story was composed of participants’ direct quotes. Further expansion would require additional researcher creative writing, which could shift the balance away from original data and risk diluting the authenticity of participants’ lived experiences. In addition, the relatively short stories (~500 words) were informed by considerations of engagement and accessibility for non-academic audiences, including our partnering organization and its members. Without expanding the stories, we discussed this length concern as a study limitation in the manuscript:  “It was also possible that these participants were more actively engaged with the Ontario Electrical League. Expanding recruitment beyond the Ontario Electrical League and identifying less-active members may provide a more comprehensive view of experiences across diverse work settings. Such expansion can also enrich the data and allow for the development of more detailed stories. The current stories were intentionally kept concise (approximately 500 words) to prioritize participants’ direct quotes. Future studies may construct longer, more detailed stories with enriched details while maintaining engagement and accessibility for non-academic audiences.”  Line 396-403 |
| Discussion |  |
| Line 290~291: This is a significant result, but I don't feel like I read anything up to this point that supports this. It's very likely in your data, but didn't make it into the paper. But, since you're discussing it, please make sure that something from your interviews is included to support this. | This statement was made based on the incident described in Story, in which Mike encountered racist remarks from a customer. We agree that the original statement might be more appropriate in describing quantitative than qualitative results. Therefore, we’ve changed this sentence and linked it to the story details presented in the results:  “Although our findings suggested that overt incidents of discrimination were not frequent, they could still have meaningful impacts on participants’ emotional and psychosocial well-being. In the current study, participants described their experiences using terms such as “pretty awful” and “shaking”. However, the emotional effects of such incidents may extend beyond these immediate reactions and could lead to long-term consequences for the mental health and wellbeing [6].”  Line 345-349 |
| Line 298-300: Do we have any data on the rate within the skilled trades? Curious if it's higher, lower, or the same. In any of these cases, worth discussing. | Thank you for the question. We added the relevant information to this section and highlighted the gap in trade-specific data:  “Canadian Apprenticeship Forum (2009) suggested that only about half of the skilled trade workers had disclosed their disability [40]. While trade-specific data remain limited, 35% of Canadian workers with disabilities never request accommodations from employers [41].”  Line 267-369 |
| Line 303-310: This is all very briefly discussed, but this sounds incredibly important. I would love to see a greatly expanded focus on these topics. | We agree that mental health is a key topic for this study. As suggested, we’ve expanded our discussion on this aspect:  “Across all three stories, participants described mental health challenges similar to those experienced by electrical workers more broadly, such as anxiety, stress, and burnout [7]. However, our findings suggested that underrepresented workers tended to rely on non-workplace support to cope with the challenges. For instance, when asked participants how they coped with mental health strains resulting from the workplace, the most frequent answer was support from family and friends. An effective social support system, however, should also include relationships at work, such as relationships with employers and with colleagues/peers [42]. Our team’s prior research has identified multiple industry-wide barriers to workers’ mental health and psychosocial wellbeing, including the societal stigma surrounding skilled trade professions, limited workplace equity and inclusiveness, and a lack of mental health resources and awareness [42,43]. This current study further identified the presence of a “toughness” culture, which had discouraged open discussion about mental health or limited their intention to seek support from the workplace. This internal culture could intersect with the societal stigma and may hinder workers from thriving in their careers, particularly for those who are underrepresented individuals [43]. Together, these findings emphasized the urgent need for cultural change related to mental health promotion within the industry. Our recommendations include refining employer-employee hierarchy, developing workplace mental health resources and social support system, and initiatives to normalize mental health conversations.”  Line 372-389 |
| Conclusion |  |
| I do not doubt that your interview data supports many or all of these conclusions, however, what was presented here does not feel sufficient to support these conclusions.  Overall, this matches up with my general impression, which is that your research questions, data, and the framework of this paper could probably be greatly expanded in order to be much more impactful. | Thank you for this thoughtful comment. In response, we have substantially revised the manuscript to better align the data presented with the conclusions drawn. Specifically, we (1) expanded the Introduction to more clearly justify the focus on the electrical trade; (2) enhanced the Methods section by adding a detailed description of the creative non-fiction methodology; and (3) expanded the Discussion section, with particular emphasis on mental health and explicit integration of findings with our team’s prior research, directly addressing the concerns raised above.  In addition, we agree that further use of participant quotes would strengthen the manuscript. We have therefore incorporated additional quotes which did not fit naturally within the stories into a new appendix. We hope these additions provide greater transparency, strengthen the evidentiary basis of our conclusions, and better demonstrate how the interview data support our interpretations. |
