# Supplementary material for: A qualitative study of lived experiences of underrepresented electrical workers using creative non-fiction
Source: PLoS One. 2026 Mar 23;21(3):e0345572. doi: 10.1371/journal.pone.0345572 (PMC13008067; doi:10.1371/journal.pone.0345572)
Supplement: Attachment — Submitted filename: Response_to_Reviewers_auresp_2.docx [file pone.0345572.s003.docx]

**Response to Reviewers**

We greatly appreciate the reviewer’s feedback on the revision. We have considered their comments and revise the manuscript accordingly. Below includes our responses and modifications.

| Reviewer comment | Response |
| --- | --- |
| **Journal Requirement** |  |
| If the reviewer comments include a recommendation to cite specific previously published works, please review and evaluate these publications to determine whether they are relevant and should be cited. There is no requirement to cite these works unless the editor has indicated otherwise. | Not applicable |
| Please review your reference list to ensure that it is complete and correct. If you have cited papers that have been retracted, please include the rationale for doing so in the manuscript text, or remove these references and replace them with relevant current references. Any changes to the reference list should be mentioned in the rebuttal letter that accompanies your revised manuscript. If you need to cite a retracted article, indicate the article’s retracted status in the References list and also include a citation and full reference for the retraction notice. | We reviewed the reference list and did not need to make any changes. |
| **Additional Editor Comments** |  |
| **Methods** |  |
| Add more detail on recruitment. How were they approached? Which method of modification of sampling? What was the study population? How many participants were approached and what was the response rate? | We’ve added additional information on the recruitment processes as requested:  “The study population included electrical apprentices, workers, and employers who self-identified as women, Indigenous peoples, racialized individuals, and/or persons with disabilities. Recruitment was conducted through purposive sampling in collaboration with the Ontario Electrical League, a provincial advocacy organization representing 38,000 non-unionized electrical workers. Eligible participants were initially contacted by the Ontario Electrical League staff via email and phone call to gauge their interest. With participants’ consent, the Ontario Electrical League shared contact information (participants’ email and/or phone number) with the research team. Response rates were not available because the Ontario Electrical League did not disclose the total number of participants contacted. Additional recruitment was carried out at the Ontario Electrical League’s chapter meetings across Ontario. The research team attended and presented the research at the meetings. Participants contacted the research team by email following the meetings. Written consent was obtained prior to data collection. Recruitment took place between November 15, 2024 and March 31, 2025. All procedures were approved by the University of Toronto Research Ethics Boards.” Line 110-123 |
| **Data collection** |  |
| Thank you for the appendix, please add detail to the structure and selection of the interview questions, and the justification behind this choice as it relates to the study aim. | We’ve explained our decision on the interview structure and the questions:  “Two shorter interviews allowed for greater rapport-building, improved data richness, and respected participants' time, while enhancing reflexivity and trust [22]. The interview guide was structured into three thematic sections to align with the research questions. The first section focused on building rapport with participants, understanding their backgrounds, and discussing their perspectives on the current workplace culture through questions such as “Can you tell me about youself?” and “How would you describe the general work culture within your organization?”. The second and third sections aimed to directly address the research questions on participants’ experiences and perspectives related to mental health and workplace integration, such as “have you experienced any mental health challenges associated with your work?”.  Line 128-146. |
| **Data analysis** |  |
| Much improved, thank you. Please add a critical science approach by adding a reflexive note on the positionality of the two researchers involved in the narrative writing to make readers aware of any positional biases or otherwise. | Thank you for the comment. We have decribed the positionality of the two researchers:  “Third, the involvement of two independent coders in the narrative thematic analysis process allowed multiple perspectives and interpretations to be acknowledged via investigator triangulation [30]. Both coders are men, racial minorities, not living with disabilities, while they have differing levels of research expertise in mental health, occupational health, and disabilities studies.”  Line 175-178 |
